# Attention deficit hyperactivity disorder (ADHD) & COVID-19: Attention deficit hyperactivity disorder: Consequences of the 1st wave

**DOI:** 10.1192/j.eurpsy.2021.1752

**Published:** 2021-08-13

**Authors:** I. Insa, J.A. Alda

**Affiliations:** 1 Child And Adolescent Mental Health Service, Sant Joan de Déu Hospital, Esplugues de llobregat, Spain; 2 Child And Adolescent Mental Health, Sant Joan de Déu Hospital, Esplugues de llobregat, Spain

**Keywords:** ADHD, Mental health, Child, adolescent, Covid-19, first wave

## Abstract

**Introduction:**

In times of pandemic as Covid-19, as in disaster situations, there is an increased risk of Post-Traumatic Stress Disorder, Depression, and Anxiety (Kaufman et al., 1997; Douglas, Douglas, Harrigan, & Douglas, 2009; Guessoum et al., 2020). The measures taken have affected individual freedoms for the benefit of community health by seeking to reduce the spread of the virus, although the side effects can cause profound damage to society, especially in those most vulnerable populations such as children and adolescents with ADHD.

**Objectives:**

This study aims to assess the psychopathological state and possible consequences of the first wave of the Covid-19 pandemic.

**Methods:**

This study is part of the Kids Corona, an institutional research project of the Hospital de Sant Joan de Déu and the Fundació de Sant Joan de Déu to provide a social and research response to the Covid-19 Pandemic. To assess the impact of Covid on children and adolescents with ADHD a cross-sectional study was conducted between 20 and 30 July 2020 with a battery of questionnaires. The Child Behavior Checklist (CBCL), the Sleep Disturbance Scale for Children (SDSC), the Screen for Child Anxiety Related Disorders (SCARED), the Children’s Depression Inventory (CDI) were used.

**Results:**

70% of children and adolescents aged 7 to 12 years with ADHD had sleep disorders, the 54% had anxiety symptoms and 9% had a depressive disorder.

**Conclusions:**

Children and adolescents with ADHD are a vulnerable population to the effects of Covid, with anxiety, depression, and sleep disorders.

**Disclosure:**

No significant relationships.

